# RACS: rapid analysis of ChIP-Seq data for contig based genomes

**DOI:** 10.1186/s12859-019-3100-2

**Published:** 2019-10-29

**Authors:** Alejandro Saettone, Marcelo Ponce, Syed Nabeel-Shah, Jeffrey Fillingham

**Affiliations:** 1 Department of Chemistry and Biology, Ryerson University, 350 Victoria St, Toronto, M5B 2K3 Canada; 2 SciNet High Performance Computing Consortium, University of Toronto, 661 University Ave, Toronto, M5G 1M1 Canada; 3 Department of Molecular Genetics, University of Toronto, 1 King’s College Cir, Toronto, M5S 1A8 Canada

**Keywords:** Chromatin immunoprecipitation, Next generation sequencing, Bioinformatics pipeline, High-performance computing, *Tetrahymena thermophila*

## Abstract

**Background:**

Chromatin immunoprecipitation coupled to next generation sequencing (ChIP-Seq) is a widely-used molecular method to investigate the function of chromatin-related proteins by identifying their associated DNA sequences on a genomic scale. ChIP-Seq generates large quantities of data that is difficult to process and analyze, particularly for organisms with a contig-based sequenced genomes that typically have minimal annotation on their associated set of genes other than their associated coordinates primarily predicted by gene finding programs. Poorly annotated genome sequence makes comprehensive analysis of ChIP-Seq data difficult and as such standardized analysis pipelines are lacking.

**Results:**

We present a one-stop computational pipeline, “Rapid Analysis of ChIP-Seq data” (RACS), that utilizes traditional High-Performance Computing (HPC) techniques in association with open source tools for processing and analyzing raw ChIP-Seq data. RACS is an open source computational pipeline available from any of the following repositories https://bitbucket.org/mjponce/RACS or https://gitrepos.scinet.utoronto.ca/public/?a=summary&p=RACS. RACS is particularly useful for ChIP-Seq in organisms with contig-based genomes that have poor gene annotation to aid protein function discovery.To test the performance and efficiency of RACS, we analyzed ChIP-Seq data previously published in a model organism *Tetrahymena thermophila* which has a contig-based genome. We assessed the generality of RACS by analyzing a previously published data set generated using the model organism *Oxytricha trifallax*, whose genome sequence is also contig-based with poor annotation.

**Conclusions:**

The RACS computational pipeline presented in this report is an efficient and reliable tool to analyze genome-wide raw ChIP-Seq data generated in model organisms with poorly annotated contig-based genome sequence. Because RACS segregates the found read accumulations between genic and intergenic regions, it is particularly efficient for rapid downstream analyses of proteins involved in gene expression.

## Background

In the last few years, traditional HPC centers, such as SciNet at the University of Toronto [1], have been witnessing the emergence of increasing amounts of work-flows from non-typical disciplines in the field of computational science [2]. Among those, disciplines related to bioinformatics appear to be the most prominent in terms of demanding resources and tackling complex biological questions an example of which related to the understanding of the mechanisms underlying transcription. Some of these biological questions are being answered by Next Generation Sequencing (NGS). For example, NGS-based methodologies are helping to address biological questions including the human genome project [3], the human microbiome project [4], RNA-Seq to analyze gene expression [5, 6] and Chromatin immunoprecipitation coupled to NGS (ChIP-Seq) to assess global DNA-binding sites [5, 6].

The advantage of these NGS methodologies for researchers is that high-throughput sequencing allows millions of DNA molecules to be read at the same time [7–9]. The output of NGS is therefore substantial and can be overwhelming for analyses [10, 11]. These analyses are facilitated in model organisms that feature well-annotated genomes such as humans and yeast where genomic sequence is presented in full chromosomal form, the DNA sequence of which can be found as individual files. These genomes have available annotation files that depict the chromosome-specific DNA base pair coordinates of cis-acting DNA sequences including, open reading frames (ORFs), untranslated regions, transcription start sites, and promoter sequences as well as information about the genes themselves taken from the scientific literature making the interpretation of ChIP-Seq data of transcription proteins more accessible. The difficulties during NGS analyses can be compounded if the genome under study is presented as contig-based (contiguous) sequence assemblies, as is the case in the model ciliates *T.thermophila* and *O.trifallax*. A contig-based genome sequence is structured and presented as a basic assembly of consensus regions based on overlapping DNA sequences obtained from DNA sequencing. Contig-based genome sequences are usually available as a conglomerate of individual contigs in a large file. These genome sequences frequently provide files with minimal annotations of predicted genes usually reflecting the lack of available information in the literature.

ChIP-Seq is used in gene expression studies to make predictions about the function(s) a protein in transcription based on its position within a gene [9, 12]. For example, if the Protein of Interest (POI) accumulates within genes rather than intergenic regions, we could infer that it might have a direct role in transcription regulation. An enrichment of the ChIP peaks near the 5’UTRs would suggest that the POI likely functions in transcription initiation. On the other hand, accumulation of ChIP peaks at the 3’ ends would suggest a role in transcription termination while proteins involved in elongation are typically found throughout the coding region. Note this is only a first approximation since gene expression can also be coordinated by elements that are not in close proximity to the specific gene [13].

To determine POI position(s) within a genome from raw ChIP-Seq data, the files containing gene coordinates are needed. It is important to note that less developed genomes such as that of *T.thermophila* and *O.trifallax* provide files containing the predicted coordinates for gene positions as minimum annotation. Current ChIP-Seq applications such as MACS2 [14] do not directly address whether the accumulation of the POI is in a specific area such as genic or intergenic region. To obtain a genome file that can be used by a software like MACS2 many other computational steps are required. After the initial alignment, the data is typically analyzed by a peak calling software, such as MACS2, which provide with peaks coordinates. The user then needs to further process the peaks obtained with third-party softwares such as BEDTools [15] to assess the local enrichment within genic and/or intergenic regions.

Our computational pipeline **R**apidly **A**nalyze **C**hIP-**S**eq data (RACS) can be used for any genome that has files containing coordinate sequences of interest. Our pipeline provides a unified tool to perform comprehensive ChIP-Seq data analysis. For instance, with RACS users obtain the co-ordinates of ChIP peaks as well as information regarding their relative enrichment across the genome, i.e. number of significant peaks found with genic versus non-genic regions. We suggest that RACS is a versatile computation pipeline suitable to analyze ChIP-Seq data generated using any model organism.

## RACS pipeline implementation

In this work, we describe and demonstrate the utility of the RACS pipeline using two ChIP-Seq data sets generated in two different model organisms including *T.thermophila* and *O.trifallax*. The *T. thermophila* ChIP-Seq data set originates from our recent study [16] on the Ibd1 protein that we found to be a component of multiple chromatin remodeling complexes and localized mainly to highly transcribed genes. Here, we used RACS to refine the Ibd1 ChIP-Seq analysis by subtracting data from an untagged control sample. The *O.trifallax* data set is derived from a study that suggests that RNA Polymerase II (RNAPII) is involved on genome-wide nanochromosome transcription during development [17]. RACS analysis gives results comparable to the reported ChIP-Seq data for *O.trifallax* RNAPII supporting the use of RACS as a generic pipeline.

The RACS pipeline is an open source set of shell and R scripts, which are organized in three main categories:

- the *core pipeline* tools, which allow the user to compute reads differentiating between genic and intergenic regions automatically
- auxiliary *post-processing* scripts^1^ for normalization using the “Cluster Passing Filtering” (PF) values
- and *utilities* to validate results by visualizing the reads accumulation and run comparisons with other software tools, such as IGV and MACS2 respectively.

**Fig. 1:**
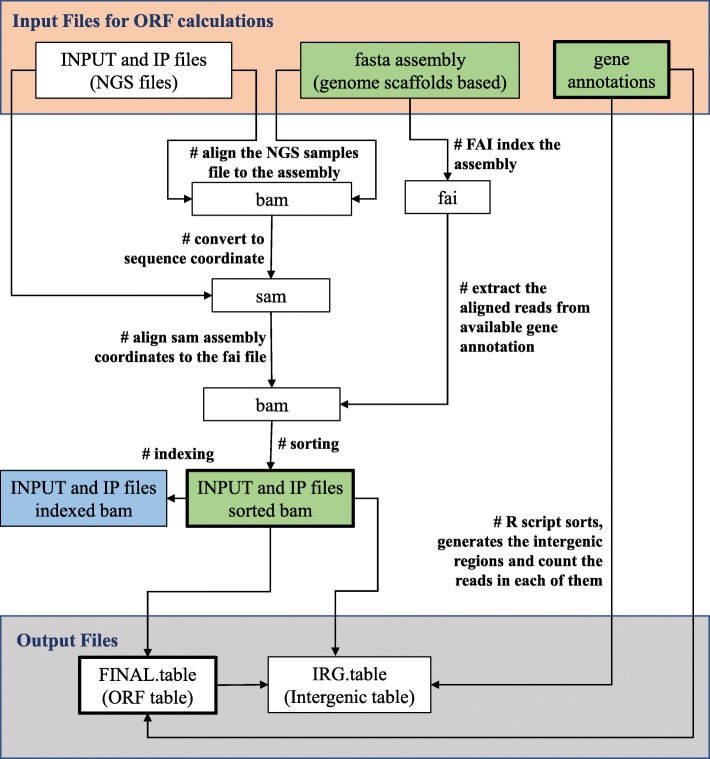
Core RACS pipeline overview. This flowchart represents the logic steps implemented in the core pipeline. Boxes represent files and file types as indicated in the text. Files with thick boxes represent the Input Files for Intergenic calculations. Files in green are to be uploaded to IGV. File in blue is needed for IGV but it does not have to be uploaded to IGV. This file has to be kept in the same folder directory than the sorted bam

The RACS repository includes the core or main scripts placed in the “core” directory. The comparison and auxiliary tools are placed in a “tools” directory. We have also included examples of submission scripts in the “hpc” directory, with PBS [18, 19] and SLURM [20, 21] examples of submission scripts, so that users with access to HPC resources can take advantage of them. Additionally, we have included a “datasets” directory containing scripts that allow the user to download the data used in these analyses. Details about the pipeline implementation and how to use it are included in the ‘README’ file available within the RACS repositories. A generic top-down overview of the pipeline implementation for the data analysis, is shown in Fig. 1.

The RACS pipeline will run in any standard workstation with a Linux-type operating system. In addition, the following open source tools are needed by the RACS core scripts:

- Burrows-Wheeler Alignment (BWA) version 0.7.13 [22]
- Sequence Alignment/Map (SAMtools) version 1.3.1 [23]
- the R statistical language [24]

Our pipeline is open source, and the scripts are available to download and accessible from public repositories^2^.

The pipeline requires as input the *.fastq* files (obtained from NGS) from the ChIP-Seq experiments and the specific genome assembly files and a file containing the gene annotations (e.g. *.gff3* files containing genic regions) corresponding to the organism.

For *T.themophila* these files are: T_thermophila_June2014.assembly.fasta and T_thermophila_June2014.gff3. Both files can be found at http://ciliate.org/index.php/home/downloads [25].

For *O.trifallax* these files are: Oxytricha_trifallax_022112_assembly.fasta and Oxytricha_trifallax_022112.gff3. Both files can be found at http://oxy.ciliate.org/index.php/home/downloads [25].

### Core pipeline tools

Our core scripts do not require any additional packages other than the ones mentioned above; however, the comparison tools, depending on what format the data to compare with is given, might use some additonal R packages, such as a spreadsheet reader package. For instance, we have included one named *.xlsx* which allows to read proprietary formats. The results of the genic and intergenic regions are generated in two *.csv* files. These are standard text ASCII files, which can be read with any typical spreadsheet software or R.

#### Determination of the genic regions

**Fig. 2:**
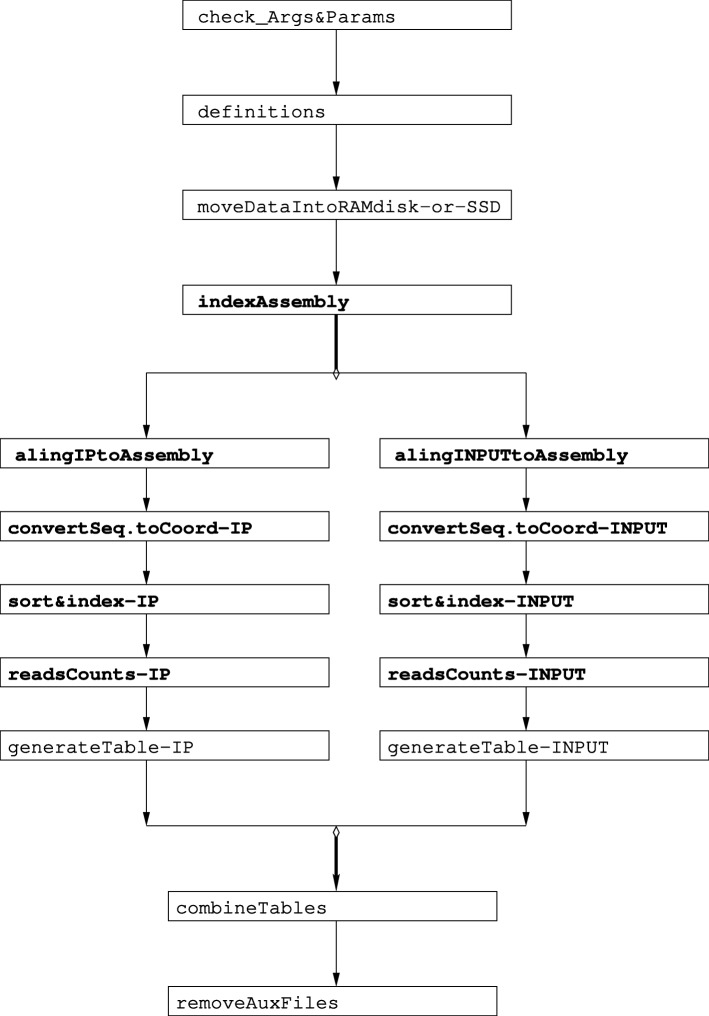
Schematic diagram of the tasks implemented for the RACS *core pipeline*. Included are details of the processing stages in relation to the scaffold based genome and the breakdown of each these steps. Bold names, indicate bioinformatic specific modules while normal fonts represent generic ones. The bifurcation represents tasks that can be executed in parallel, as there is no data dependency among them

To count the amount of reads in each genic region the core pipeline script was implemented using Linux shell commands combined with the usage of BWA and SAMtools. The input files are the genome of reference (T_thermophila_June2014.assembly.fasta), the gene annotation file (T_thermophila_June2014.gff3) and the INPUT and IP files obtained from NGS. The INPUT files contain the information obtained from NGS prior to the immunoprecipitation; thus, this file contains the initial reference amount of DNA reads. The IP file contains the data after the immunoprecipitation; thus, this file contains the DNA that were enriched by the POI. After the INPUT and IP sequences are aligned with the genome and sorted, the script uses a loop to count the reads in each genic region and deposits the obtained data in a file named “FINAL.table.*INPUTfile*-*IPfile*”; where *INPUTfile* and *IPfile* are the INPUT and IP files respectively. Figure 1 depicts a flowchart representing the required steps to obtain the final table containing the number of reads found in each of the genic regions. Details of the processing stages are shown in Fig. 2, in relation to *T.thermophila* scaffold database and the breakdown of each these steps.

The RACS pipeline was implemented to specifically target data from the *T.thermophila* organism in particular utilizing an specific *gff3* file. However due to the modular fashion in which RACS was implemented, it is possible for users targeting different organisms and even different markers, to “instruct” RACS to do so. At the level of the IGR, if the reference file follows the usual gff3 structure, nothing has to be modified in the pipeline. As a matter of fact, we implemented several checks in order to verify and guarantee the consistency of the data provided by this file. At the level of the ORF, the user will need to specify a few parameters that will be used when the targets depart from the ones used by default in the pipeline. The terms and filters allow the user to target either genes or mRNA or any other specifier within the reference file, making essentially agnostic of the organism type. In order to achieve this, the user should provide a ’definition’ file, indicating the targets for the pipeline for which to filter for the reference file. We have included a subdirectory in the repository "core/defns", where we include some files exemplifying the implementation of different cases and organisms.

In particular, the variables filter1, filter2, as well as, delim1, delim2, delim3; should be adjusted correspondingly to the organism of interest and the way the data is organized within the reference file. The following code shows an example of how this is done for *T.thermophila* and *O.trifallax*.


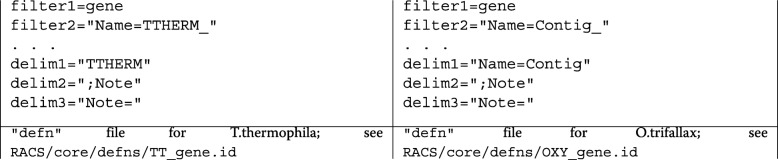


#### Determination of the intergenic regions

The intergenic regions were not available neither determined by the standard packages. For this reason, we developed an R script to determine these regions. In this pipeline these sequences are calculated during each run to account for further genome actualizations. The inputs for this script are the files generated by the genic regions pipeline discussed in the previous section (i.e. “FINAL.table.*INPUTfile*-*IPfile*”, the INPUT and IP *.bam* files –which are generated as intermediate files of the Genic Region pipeline–) plus the gene annotation file (e.g. T_thermophila_June2014.gff3). First, the script determines the intergenic regions by calculating the beginning and end of each annotated gene within each available scaffold and subtracts these values. The algorithm only reports intergenic regions that are equal or greater to zero. In the earlier version of the pipeline this constraint was not included, and in some cases, it could result in the pipeline reporting regions with negative sizes. We noticed that 92 of these cases were presented in our previous study [16]; however, we should emphasize that there were not reads present in these regions thus did not affect these results. Second, the script uses the newly generated intergenic regions to count the number of reads in each of them. Finally, the data is deposited in the intergenic table for each of the intergenic regions Fig. 1.

### Post-processing

#### Normalization of reads accumulation and enrichment calculation

To account for differences in the amount of clusters PF (reads) presented among samples, each of the obtained INPUT and IP values were normalized by dividing them by the corresponding clusters PF value of the Flowcell summary (obtained from the NGS run) or from the Total Sequences (obtained from the fastQC file). These calculations can be done by the script “normalizedORF.sh” located in the core directory of RACS. Alternatively, it can also be calculated employing the following two spreadsheets: for the genic regions (*TET_Ibd1_MAC_Genome_Genic.xlsx*), and for the intergenic regions (*TET_Ibd1_MAC_Genome_Intergenic.xlsx*); that can be found in the “datasets” subdirectory within the RACS repository. Notice that there are several spreadsheets provided in this subdirectory, each of them will be used for different organisms/cases and can be used as templates for other datasets.

These spreadsheets contain the reads found in the untagged (or mock purification/negative control) samples in the *Untagged* tab. The user can also add the Flowcell summary details in the *Add_FCS_for_(SAMPLE_ID)* tab. The user can manually introduce the read values for the samples being analyzed in the *Add_(SAMPLE_ID)_ChiP_Seq* tab. In this tab the user can divide the number found by RACS by the corresponding cluster PF number found in the previous tab. This data can be deposited in the “Normalized_INPUT or _IP (FCS)” columns. After the required reads normalization, the accumulation can be obtained as the number of IP reads divided by the number of INPUT reads (IP/INPUT). This can be deposited in the “Enrichment_(N_IP(FCS)/N_INPUT(FCS)” column of the same tabs. The obtained values are filtered (*Filter 1*) by the user by subtracting the corresponding number found in the *Untagged* tab and deposit the values in the *Enrichment_Minus_AVERAGE_untagged* column. If there are more than two samples the values can be averaged and values that are less than 1.5 can be filtered (*Filter 2*) and deposited in the “Enrichment_Average_Sample” tab. For the genic region table, in this tab there is a column containing the Expression profile obtained from the *RNA_Seq* tab. We recommend to copy the filtered cells to the *Results* tab. The distribution of the protein of interest can be calculated in this tab. For the Intergenic table there is a *ORF_vs_IGR* (Intergenic) tab where the number of regions and reads can be calculated. The number of regions is represented by the number of genic and intergenic regions that passed the 2 filters. If there is data available for untagged samples, please refer to the “Utilities: validation and quality checks” section.

The number of reads found in the genic and intergenic regions can be calculated by adding all the available values from the “Normalized IP (FCS)” columns and deposit them in the *ORF_vs_IGR* tab of the Intergenic table.

During the post-processing steps, it is important to note that some regions presented in the processed table may have very few reads after subtracting the values obtained from untagged samples and they may seem as real interactors when they are not. For instance, a sample that has 2 reads in the INPUT and 10 reads in the IP will return an enrichment of 5 and it may pass the filter of 1.5 × enrichment but they may not be significantly enriched.

### Utilities: validation and quality checks

To account for biological and experimental variability in the wet lab, we typically perform ChIP-Seq using 2 independent samples for each distinct strain and average their Enrichment. To validate the findings, it is important to determine the genic and intergenic regions of the untagged (negative control) INPUT and IP samples. After this determination, we subtracted the obtained average enrichment from untagged to the obtained tagged average of the samples. Then we filtered for values that had an enrichment greater than or equal to 1.5 in the final enrichment column. These are the enriched regions and represent genomic regions to which the POI binds.

#### Visualization of reads accumulation

The browser IGV [26] can be used to visually inspect and validate the obtained reads based on their ranked enrichment. The files needed are illustrated in Fig. 1 and the ‘README’ file included in the RACS’ repository. MACS2, a main-stream application to call peaks, can also be used as specified in [14]. MACS2 uses the same intermediate files (*.bam*) obtained from the RACS pipeline, hence it can be a good reference to be considered for comparison purposes.

### RACS performance

RACS can be run in any normal Linux workstation; however, it can also take advantages of cluster-type environments. In particular, several stages of RACS can be run using multicore architectures with several threads in parallel. In addition to that, RAMdisk can be used to speed up file I/O operations. This is achieved by indicating thorugh a command line argument the specific location for the “working space” that RACS will use to place the input and temporary files to be generated. When we originally developed our pipeline, we tested it in our previous HPC cluster, GPC [1] consisting of 2.53 GHz Intel Xeon E5540, with 16 GB RAM per node (2 GB per core). By comparing the performance of RACS with a typical workstation we noticed a speed-up factor among 8 to 12 ×. We have also run our pipeline in our newest cluster, Niagara [27], of 1500 Lenovo SD350 servers each with 40 Intel “Skylake” cores at 2.4 GHz. Each node of the cluster has 188 GiB / 202 GB RAM per node, for which we have obtained a speed up of 5 to 10 ×. In other words, the whole processing of genic (ORF) and intergenic (IGR) for a typical INPUT/IP sample, took between 1 and 2 h. In addition to that, in our new system is possible to bundle 40 (80 using multithreading) processes together.

Moreover, this first release of RACS utilizes the basic SAMtools and BAM codes, however it has been reported that improvements in processing SAM files could be achieved using SAMBAMBA [28]. One of the many advantages of dealing with an open source, modular pipeline like this, is that it allows interested users to explore this possibility as well, just by modifying the tool to process SAM files and selecting the one desired.

As mentioned above, one additional functionality that RACS offers is the ability to specify the “working space”. When using the main script for counting reads in ORF, the user has the ability of indicating whether to use a faster “working space” than traditional spinning disks (ie. HDD) such as memory (ie. RAMdisk) or a solid state device (SSD). In general, utilizing RAMdisk or SSDs, would result in a speed-up of roughly 10 to 30%, depending on hardware specifications and the size of the dataset to be analyzed. The larger the dataset the more I/O operations (reads/writes) that would be needed, hence larger datasets would benefit the most of this. This is of course, assuming that the data and subsequent auxiliary files created during the analysis will fit in “memory”. If that is not the case then depending on the system and how it is configured may result in decremental performance (e.g. some computers will begin *swapping* data –i.e. start using traditional HDD space–) or even crash (for instance, is common in many HPC clusters to do not allow for swapping techniques). Differences in performance among SSD vs RAMdisk, are almost negligible, again depending on hardware specs, this can be upmost of the order of few percentages. Finally, it should be noticed that by using RAMdisk (i.e. memory) as a working space, users will reduce the overall computational time, however this is will ultimately depend upon the amount of memory available as this technique will clearly increase the utilization of RAM. As a general estimate, at the moment of running the pipeline, users might estimate the amount of memory needed by one order of magnitude larger (i.e. ×10) than the size of the dataset to be processed. Further details about memory utilization and walltimes as function of number of threads or cores, are presented in Table 2 and in the “doc” directory of the RACS repository.

## Results

In this paper we introduce a one-stop methodology to analyze ChIP-Seq data to find the set of genome coordinates for a given POI. This methodology utilizes open-source tools such as BWA [22], SAMtools [23], Linux shell and R scripts [24] and techniques commonly employed in the HPC fields. RACS can be run either in a typical workstation or taking full advantage of HPC resources, such as, multicore architectures and use of RAMdisk, to improve the analysis times making it more efficient, (see details on “RACS performance” section). This pipeline was developed to answer whether the POI localized to a given set of cordinates (genes) or to the remaining regions in the genome that were not given by the user (intergenic). RACS was designed in a user-friendly manner to accommodate researchers with basic knowledge in Linux shell and R [24]. RACS provides accessible downstream analyses of ChIP-Seq data obtained from Illumina instruments. RACS follows a unique approach to tackle this problem, is widely applicable and useful enough to analyze ChIP-Seq related data from a variety of different organisms generated by NGS.

The RACS pipeline, Fig. 2, offers a solution that utilizes an available contig-based genome sequence file and a second annotation file that contains the coordinates for the annotated genes. After processing ChIP-Seq data, RACS will output two tables, the first containing all found reads accumulation in the genic region corresponding to the annotated genes and the second containing the accumulation of reads in the intergenic regions. An intergenic region will be calculated as a region that starts at the end of a given gene coordinate and ends at the beginning of the next contiguous given gene coordinate. RACS will calculate the beginning of a contig as the beginning of an intergenic region (as long as there is not an annotated gene at the beginning of the contig) which ends at the coordinate of the first encountered gene, and it will do the same at the end of each contig. These intergenic or adjacent regions are newly generated each time to account for modifications or improvements in the files containing gene annotations. The obtained results from both tables are normalized to the number of clusters that passed Illumina’s “Chastity filter” also called clusters PF. These numbers represent the reads obtained per sample. The normalized values are further filtered by using the data obtained from the mock samples.

### Case study

**Fig. 3:**
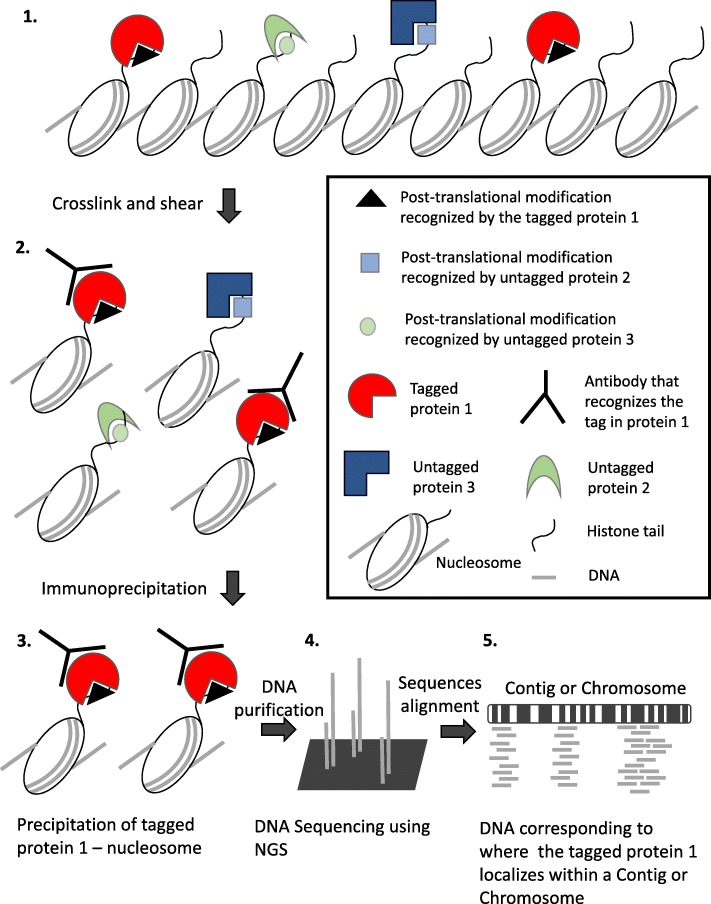
Diagram summarizing the *ChIP-Seq* technique used to prepare the samples and generate the data from the “wet-lab”: 1) Native state of chromatin. 2) Specific antibodies recognize the tagged proteins. 3) Isolation of tagged protein plus its interacting chromatin. 4) After DNA purification and library preparation NGS is performed. 5) The output data from NGS is aligned to *Tetrahymena thermophila*’s genome assembly

In this section, we describe how RACS was used to analyze and generate the data presented in [16] in addition to its refinement by introducing ChIP-Seq data from untagged strain. The model organism used in [16] is the protist Alveolate *T. thermophila* which is the most experimentally amenable member of this taxonomical group. *T. thermophila* can be used in some cases to understand the basic biology of the parasitic and disease-causing members of the Alveolates. Members of *Plasmodium* species that causes malaria [29–31] and other related species that affect ecosystems [32] and aquiculture [33] can be examined by analogy through our selected model organism. In addition, *T.thermophila* has genes that present homology to human genes [34, 35] and characteristics that makes it an excellent candidate to study chromatin mainly because of the segregation of transcriptionally active and silent chromatin into two distinct nuclei, macronucleus (MAC) and micronucleus (MIC) respectively [36]. In our recent study we identified a protein, Ibd1, that physically interacts with several chromatin remodeling complexes [16]. The *T.thermophila*’s genome [25] is contig based and contains almost 27 thousand annotated genes or genic regions [37]. To further the understanding of Ibd1, and to contribute to current understanding of how chromatin remodeling works, we analyzed its localization within the genome by ChIP-Seq (Fig. 3) [38–41]. This allowed us to identify the set of genes bound by Ibd1 to begin to understand its function.

#### Pre-processing of the fastq files and quality assessment

The ChIP samples were processed as described in [16] to make the library preparation using the TruSeq ChIP-Seq kit (Illumina). For the untagged (this study) and Ibd1 [16] strains, libraries were sequenced using the v4 chemistry in a HiSeq2500 instrument (Illumina) set for High Output mode. The obtained read lengths were of 66 base pairs, 6 base pairs corresponded to the adapters for demultiplexing. These files were demultiplexed in *fastq* format and the adapters were trimmed using the bcl2fastq2 Conversion Software v2.20.0. The obtained *fastq* files for the INPUTS and IP samples were assessed by fastQC version 0.11.5 [42]. Each dataset obtained from the ChIP-Seq experiments has a sequence of whole cell DNA (INPUT) and DNA sequenced from an immunoprecipitated (IP) sample. The Ibd1 NGS data generated in [16] can be found at the following Gene Expression Omnibus (GEO) link: GSE103318 [16]. In addition, untagged *T.thermophila* fastq files were generated and they can be found at the following GEO link: GSE125576.

We recommend assessing the quality of the data obtained from the NGS. This step is important to have general information regarding the run. This fastQC report also helps to understand the alerts present in each sample, these alerts do not necessarily mean that the NGS run failed [42]. In other words, this step is to verify if the *fastq* data has any alerts that have to be addressed before proceeding to the processing. For example, in our case our data for the *Per base sequence quality* fell into the very good quality reads (green) area of the y axis allowing us to avoid quality trimming. On the other hand, we obtained a flag for *Overrepresented sequences*, in particular the one that called our attention was the sequence containing only the nucleotide N. Since our reads are 35-58 base pairs long, the allowed maximum mismatch to the genome will be up to 3 base pairs according to the BWA algorithm [22] hence it will not consider these sequences for the alignment.

#### Visualization and list of reads

**Fig. 4:**
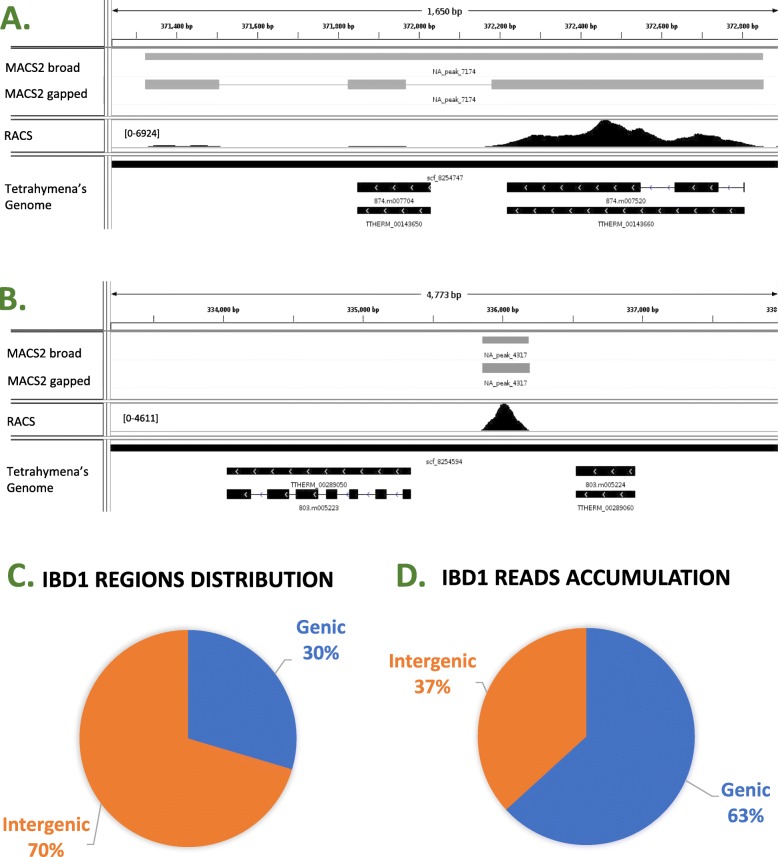
**a** and **b** Visualization genic and intergenic region using IGV. The top track shows MACS2 broad and gapped peaks. The middle track shows RACS visual representation of reads accumulation. Note that RACS shows graphical reads behaviour and accumulation preferences. The bottom track shows *T.thermophila*’s genes. On the other hand, MACS2 found two weak peaks that can be interpreted as background by our pipeline. The range inside the brackets represents the highest number of reads for that specific track. **c** Ibd1 localizes to more intergenic than genic regions. **d** The majority of reads are found in genic regions. These results take into consideration the updated information provided by the mock samples

After, the determination of enriched regions we can further analyze them using a visualization tool, such as IGV. The region of interest can be copied from either the genic or intergenic table. This localization corresponds to where the protein of interest is localizing with respect to annotated genes (see Fig. 4 panel A) or an intergenic region (see Fig. 4 panel B). It is important to note that for Ibd1’s ChIP-Seq [16] data we also used MACS2 a main-stream application to call peaks [14]. The visualization option for MACS2 and RACS are similar in that both provide a specific file that can be used for this purpose. In the case of RACS, our pipeline uses *.bam* and *.fai* files which are generated within the GENIC part of the pipeline (see Fig. 1). Such *.bam* files can be opened in IGV, although the *.fai* (index) file will not, however both files should be present in the same directory. The required files for IGV visualization are depicted in Fig. 1. In addition, the *.bam* files generated by RACS can be used as input for MACS2. When compared the MACS2 visualization file to the RACS *.bam* files using IGV (Fig. 4, panels A and B), we observed that the RACS files provide a visual of the portion enriched. Here we observed that the IP samples are clearly enriched regions showing peaks when compared to the INPUT samples. This can be determined by noting the numbers shown in each of the IGV tracks, which represent its corresponding reads accumulation.

The track corresponding to our pipeline in Fig. 4 panel A shows clear accumulation of reads on the gene that is on the right side as it is in the track form MACS2. However, for the gene and the intergenic region on the left side of the track RACS does not show a clear accumulation whereas MACS2 does. Figure 4 panel B shows a perfect match. The ability of comparing these two tools at the same time can help the user by providing more robust results that can lead or not to further investigation of the specific sequence.

The output lists provided by RACS are segregated into two *.csv* files. The first file contains the genic and the second the intergenic regions. Both lists contain the all reads obtained from the INPUT and IP samples. This obtained data should be filtered with the data obtained from Mock samples. We found that the output of MACS2 provides a list of peaks. MACS2 does not classify the peaks based on the localization to a gene or an intergenic region as RACS does. However, this can be addressed using BEDTools [15] after MACS2 analysis. The datasets generated by MACS2 and RACS can be found at the GEO link GSE103318.

#### Mock samples facilitate the analysis

In [16] we found that the majority of genes regulated by Ibd1 were highly expressed housekeeping genes. For that analysis a filtering step using ChIP-Seq data from untagged samples were not employed, and instead a cut-off was implemented based on accumulation of reads. However, since the cut-off was arbitrary, there was some degree of uncertainty in regards of its astringency. To overcome this limitation and further facilitate the analysis, for this study we performed a mock ChIP-Seq experiment using untagged control *T.thermophila* cells in order to reveal the identity of the set of specific DNA sequences that have affinity for the antibody-conjugated chromatography resin either directly or mediated through unknown protein(s) in the chromatin extract. RACS analysis of the Ibd1 ChIP-Seq dataset filtered by two mock IP ChIP-Seq replicas from untagged *T.thermophila* enhanced RACS’ ability to discriminate non-specific DNA binding (see “Post-processing” section Post-processing for further details). In addition, the use of mock ChIP-Seq samples eliminated the uncertainty associated with using the arbitrary cut-off. Between both the analysis presented in [16], and this new analysis (RACS), there are not major statistical differences regarding Ibd1 localization to genes that are highly, moderate, or low to no-expressed (Table 1). The statistical analysis presented in Table 1 shows that the hypothesis generated in [16] regarding an Ibd1 function related to transcription of highly expressed genes stands. The calculation of this result can be found in the Result tab of the genic table (RACS/datasets/TET_Ibd1_MAC_Genome_Genic.xlsx ).

**Table 1:** Comparison of Ibd1 localization presented in [16] (without untagged controls) and analyzed by RACS using untagged controls (current study)

(a) Ibd1 localization presented in both studies.		
	Ibd1 localization %	Ibd1 localization %
Expression level	using untagged controls	without untagged controls
High expression	51	54
Moderate expression	6	16
Low to no-expression	16	14
Non-available expression		
for the TTHERMs in the		
GSM692081 data set	27	16
(b) t-Test: Paired Two Sample for Means		
t-Test: Paired Two Sample for Means		
	Ibd1 localization % using untagged controls	Ibd1 localization % without untagged controls
Mean	25	25
Variance	374	374.6667
Observations	4	4
Pearson Correlation	0.89581514	
Hypothesized mean difference	0	
df	3	
*t* Stat	0	
*P*(*T*≤*t*) one-tail	0.5	
*t* Critical one-tail	2.35336343	
*P*(*T*≤*t*)two-tail	1	
*t* Critical two-tail	3.18244631	

There is a correlation of 0.896 and non-statistical differences between the two data sets. The data presented in [16] uses an arbitrary cut-off. The data presented in this paper does not use the arbitrary cut-off and instead uses as cut-off the values obtained by the analyses of the untagged samples

#### RACS aids in the determination of the protein of interest function

To gain insights in the POI function, we segregated its localization between genic and intergenic. After analyzing Ibd1’s raw ChIP-Seq data with RACS, tables with the total number of reads found in each of the 26,996 genic and 27,780 intergenic regions were generated [16]. From the genic and intergenic tables we observed that Ibd1 localizes to more individual intergenic regions than genic regions (Fig. 4 panel C). However, the majority of reads accumulation are in the genic regions (Fig. 4 panel D), suggesting that Ibd1 primary localization is within the genic implicating Ibd1 function in transcription regulation.

**Fig. 5:**
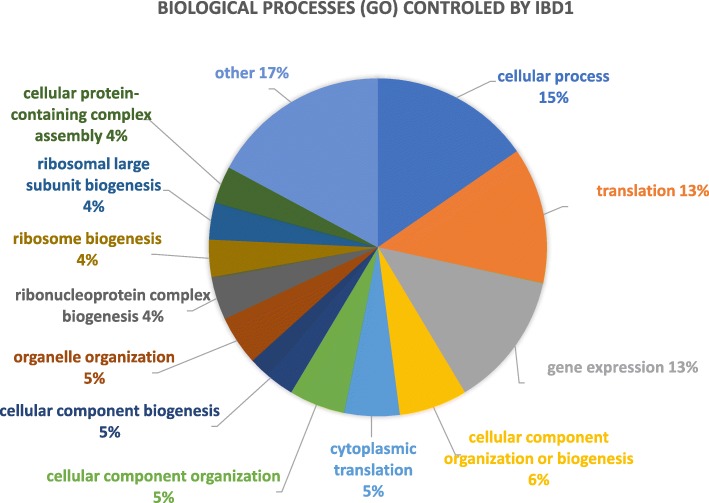
Gene Ontology (GO) analysis of genes controlled by Ibd1. GO predicted that the majority of Ibd1 bounded genes are related to housekeeping functions

The function of Ibd1 was further inferred based on the GO annotations for biological process [43] categories of genes to which it binds. From the genic table (RACS/datasets/TET_Ibd1_MAC_Genome_Genic.xlsx ) we observed that Ibd1 mostly localizes to genes that are highly expressed and related to housekeeping function; such as, cellular function, translation, gene expression, biogenesis, cytoplasmic translation among others (see Fig. 5). The calculation of this result can be found in the *Gene_Ontology* tab of the genic table (RACS/datasets/TET_Ibd1_MAC_Genome_Genic.xlsx} ).

### RACS for *T.thermophila*’s rDNA minichromosome

The obtained data from Ibd1 ChIP-Seq was used against the rDNA minichromosome sequence [44]. Ibd1 is not enriched in any of the 3 genic or 4 intergenic regions. The generated tables can be found in the repository, under the datasets subdirectory: *TET_Ibd1_rDNA_Genic.xlsx* and *TET_Ibd1_rDNA_Intergenic.xlsx*.

#### Outliers

During the Post-processing stage, we found a great number of reads for the following three genic regions: TTHERM_02141639, TTHERM_02641280, TTHERM_02653301; in the tagged and untagged ChIP-Seq samples. After applying the 1.5 × cut-off for enrichment of Ibd1 we found that the first two mentioned genic region were filtered out after the subtraction step described in the (“Utilities: validation and quality checks” section). The third gene passed the control, thus, seems that Ibd1 localizes to this region. In other words, the accumulation of the first two regions are due to nonspecific binding and the third to specific binding. This is another example of why the untagged strains can help to determine if this found accumulated DNA in the untagged and tagged samples are or not due to specific binding.

### Performance

By implementing this pipeline as described here, we obtained roughly a factor of 4× faster in comparison to a serial and non-I/O optimized (i.e. not using RAMdisk), in an equivalent hardware to the node used in the cluster. This is something we have also observed by using similar techniques (e.g. RAMdisk) in other type of bio-informatics pipelines where the hierarchy of the computational scales is dominated by the I/O parts of the code. Moreover, we processed a second set of data, that was roughly 3 times larger than the original data –which would not fit in memory (>64 GB)–, utilizing a more modern node (i7 core) with a solid-state device (SSD), we were able to further reduce the processing time approximately by another factor of ∼4. This type of trend is typical in cases where performance is dominated by computations and I/O operations (e.g. reading and writing files), for which the combination of faster processing plus faster access to the data is essential for improving the overall performance. Nevertheless, we should emphasize that even when RAMdisk or an SSD can be a solution that could in principle be thrown to similar type of problems, i.e. intensively I/O demanding ones, the best approach would always be to try to mitigate and reduce as much as possible the I/O operations, as these usually represent the slowest part in any computational implementation.

Other points to notice are: i) in most of the cases, increasing the number of cores, improves performance in terms of speed-up factors; ii) speed-up factors, also depend on the size of the data sets, although in general they follow a very similar trend; iii) larger data sets require larger processing times, while –in general– smaller data sets show better scaling performance, which in principle can be understood as the pipeline has no communication parallelism implemented; iv) there are limitations to these scaling trends, for instance when the amount of data/work to be splitten is not big enough with respect to the overhad cost of organizing the work distribution (an example of this can be seen in Table 2 with the MED31-1 dataset when attempting to run with 64 cores).

**Table 2:** RACS scaling and performance trends for the ORF part of the pipeline: we performed the standard *strong scaling* analysis, as well as a function of different dataset sizes

Initial data size	Number of procesors	Workspace usage	Walltime time
≈3 GB^a^	1	≈27 GB	7037 secs
	2	"	5059 secs
	4	"	3856 secs
	8	"	3238 secs
	16	"	2940 secs
	32	"	2801 secs
	64^b^	"	2463 secs
≈2.4 GB^c^	1	≈20 GB	5477 secs
	2	"	4005 secs
	4	"	3128 secs
	8	"	2678 secs
	16	"	2456 secs
	32	"	2344 secs
	64	"	2161 secs
≈6.8 GB^d^	1	≈50.3 GB	6987 secs
	2	"	5662 secs
	4	"	4864 secs
	8	"	4451 secs
	16	"	4245 secs
	32	"	4148 secs
	64	"	4155 secs
≈7.1 GB^e^	1	≈53.4 GB	7728 secs
	2	"	6191 secs
	4	"	5255 secs
	8	"	4740 secs
	16	"	4529 secs
	32	"	4413 secs
	64	"	4249 secs
≈1.4 GB^f^	1	≈8.3 GB	2874 secs
	2	"	1796 secs
	4	"	1218 secs
	8	"	920 secs
	16	"	773 secs
	32	"	702 secs
	64	"	639 secs

^a^Ibd1-1 data set for *T.thermophila* [16].

^b^Although there are 40 physical cores in the TDS/Niagara nodes, *hyperthreading* is enabled so it can be used up to 80 logical cores.

^c^Ibd1-2 data set for *T.thermophila* [16].

^d^MED31-1 data set for *T.thermophila* [48].

^e^MED31-2 data set for *T.thermophila* [48].

^f^Data set for *O.trifallax*.

As it can be seen, the working space (in this case *memory utilization*) can reach up to a factor of 9-10 × the size of the initial data to be processed. Further details about memory consumption can be found in the README document and the “doc” directory, included within the RACS repository. These tests were run in the TDS system (i.e. one Lenovo SD530 node with 40 cores and 192GB of RAM with CentOS 7.4 operating system) of the Niagara supercomputer [27], utilizing RAMDISK as working space

### RACS for *O.trifallax*

**Fig. 6:**
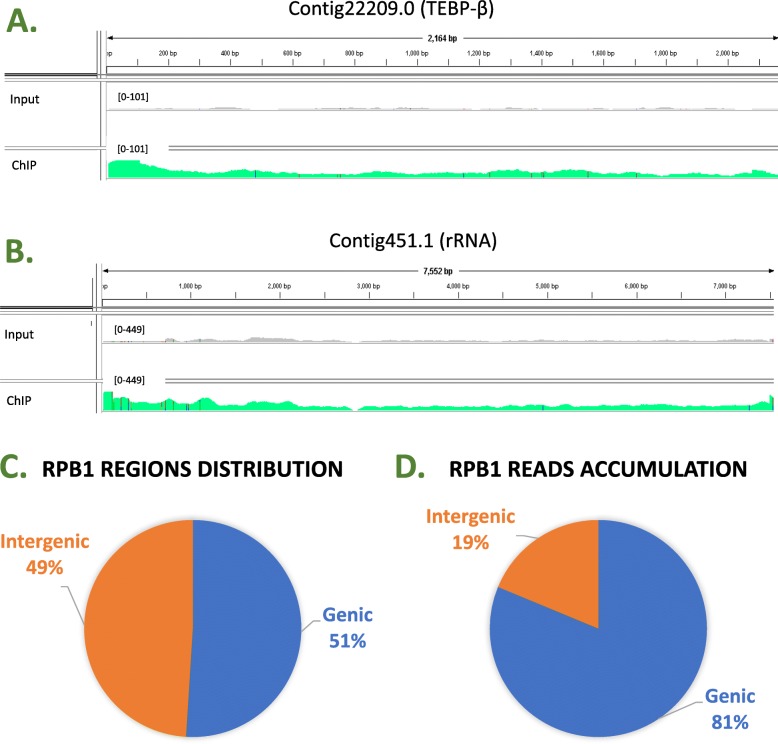
RACS analysis using *Oxytricha trifallax* ChIP-Seq Rpb1 data gives results comparable to the reported in [17]. **a** and **b** Rpb1 enriches along Contig22209.0 and Contig451.1. The range inside the brackets represents the highest number of reads for that specific track. **c** Rpb1 can be found binding a similar amount of genic and intergenic regions throughout the genome; however, **d** most of the reads that were pulled down by Rpb1 are within genic regions

To test RACS in a different model organism we utilized the data generated for the following study [17]. The used data set can be found at the GEO link: GSE55703 and the tables generated by RACS are available in the repository, under the datasets subdirectory: *OXY_Rpb1_MAC_Genic.xlsx* and *OXY_Rpb1_MAC_Intergenic.xlsx*. After analyzing this published data, we found that Rpb1 binds to 90% of the 24,885 annotated genes and to 54% of the 43,326 RACS generated intergenic regions. This result concludes that Rpb1 has a genome wide distribution and it is consistent to what it was published previously. In Fig. 6 (panels A and B) it is shown that the DNA distribution throughout the gene is consistent to what it was found in [17]. A new result found by RACS for this study is presented in Fig. 6 panels C and D. Figure 6 panel C shows that Rpb1 interacts with the same amount of genic and intergenic regions. However, Fig. 6 panel D shows that 81% of all reads are distributed in the genic regions. This shows that Rpb1 has a preference for gene bodies.

## Discussion

In this paper, we have presented RACS, a pipeline implementation utilizing open source tools for the rapid analysis of ChIP-Seq data for a POI from an organism with a contig-based genome sequence. RACS utilizes the predicted gene coordinates and groups the reads accumulation for genic and intergenic regions in ranked form. The objective is to be able to infer POI function based on its chromatin occupancy. This pipeline has been applied to *Tetrahymena thermophila* and *Oxytricha trifallax*’s ChIP-Seq data, but its application can be extended to ChIP-Seq datasets generated in any other organisms.

Initially, when we called peaks for Ibd1 ChIP-Seq data using MACS2, the output did not indicate whether the protein localizes to a genic or an intergenic region. MACS2 calls all peaks regardless of their position in a genic or intergenic region, which makes interpretation difficult when combined with the minimal annotation of the *Tetrahymena* genome. RACS segregates ChIP-Seq ranked peaks between genic and intergenic which can help to quickly assign biological function to a POI. We note that other programs, such as BEDTools, can be used to perform this task in combination with MACS2. Without the need of any additional “external” software, RACS calls peaks and segregates them in two tables based on the given set of coordinates (genes) or the remaining regions in the genome that were not provided by the user (intergenic). Thus, our pipeline is appropriate to address biological questions regarding function based on genome position.

We hold the opinion that MACS2 and RACS are complementary to each other, but empathize that they are not dependent on each other for analysis. For example, MACS2 can be used to establish or to generate a set of coordinates for a specific transcription protein binding to the genome. Thus, we can infer that the POI is attaching to specific areas in the genome to control transcription and we could annotate these regions as binding sites for the specific transcription protein. Then, if we perform ChIP-Seq on a different protein that has also been shown to physically interact with the transcription protein previously mentioned, we could use the coordinates given by MACS2 to generate a *.gff3* file to input it alongside the genome file to the RACS pipeline. This will allow us to rapidly determine the degree of overlap and potential co-localization in some or all binding sites. In that, MACS and RACS can synergize to provide a powerful tool for the analysis of less developed genome sequences.

Even when there are many computational tools available for processing ChIP-Seq data, RACS is particularly suitable for the analysis of contig-based genome sequence with associated minimal annotation. Other tools, such as, MACS2 and metagene using deepTools analysis [45] complement RACS. Recently several ChIP-Seq studies [46–48] have emerged for *T.thermophila*. However, there is a lack of standardized computational methods for this model organism, hence it becomes difficult to reliably reach at the same conclusions when replicating the findings. Our tool is the first effort in *T.thermophila* to provide a community resource for genome-wide ChIP-Seq studies, therefore it has the potential to contribute to standardization of ChIP-Seq analyses in ciliates. We intend to continue refinement of RACS based on community need. For example, recently, single-molecule sequencing based on nanopores has emerged as a promising technology with a potential to revolutionize the genomics [49]. The nanopore sequencing provides the advantages of 1) long reads, enabling the de novo transcriptome analysis [50], 2) point-of-care, making real-time analysis possible [51], and 3) PCR free, allowing the direct identification of epigenetics [52]. Considering its promising outcomes, studies using model organisms with divergent genomes, such ciliates and parasitic organisms including *Trypanosoma*, will be particularly benefited from the nanopore sequencing technology [53]. Currently, a major challenge is to develop sophisticated and high-performance computational tools to interpret and analyze the nanopore sequencing data [54–56]. In future, we aim to improve and implement the RACS pipeline for the analysis of nanopore sequencing data.

## Conclusions

RACS is an excellent tool for genomes that are contig-based and/or have poor annotations, it permits the segregation of reads accumulation between genic and intergenic region after ChIP-Seq processing. RACS is complementary to other tools, such as MACS2, as it can help to discriminate complex regions improving the overall analysis.

RACS offers an alternative tool with a different approach focused on a simple, modular and open approach. RACS offers a versatile, agile and modular pipeline that cover many of the steps needed in the process of analyzing ChIP-Seq data.

The pipeline uses HPC tools, such as RAMdisk or batch processing via scheduling in cluster type environments, so that the data analysis can be done for large datasets. The scripts are reusable and generic enough that can be simply modified and utilized in other pipelines as well.

The modular approach we followed when developing RACS, also allows for future developments as this pipeline could be easily ported as a backend of a web interface, or a *gateway* portal, serving a larger group of researchers from different disciplines.

## Abbreviations

ChIP-Seq: Chromatin immunoprecipitation coupled to next generation sequencing
ENCODE: Encyclopedia of DNA elements
FCS: Flowcell summary
GEO: Gene expression omnibus
GO: Gene oncology
GPC: General purpose cluster
HDD: Hard disk device
HPC: High performance computing
I/O: Input/output
IGR: Intergenic region
IP: Immunoprecipitated
MAC: Macronucleus
MIC: Micronucleus
N: Normalized
NGS: Next generation sequencing
ORF: Open region frame
OXY: Oxytricha trifallax
PCR: Polymerase chain reaction
PF: Passing filtering
RACS: Rapid analysis of ChIP-Seq data
TET: *Tetrahymena thermophila*

## Footnotes

1 Alternatively, we have also included an *auxiliary spreadsheets* that could be used instead of the script to perform the post-processing and normalization manually, as well as, to check against negative controls.

2 Both repositories are synchronized, so that the latest version of RACS is available and can be obtained from both: https://gitrepos.scinet.utoronto.ca/public/?a=summary&p=RACS or https://bitbucket.org/mjponce/RACS
